# Exploring the Impact of the COVID-19 Pandemic on Patients with Type 2 Diabetes and Primary Care Utilisation: A Scoping Review

**DOI:** 10.3390/healthcare14172804

**Published:** 2026-09-01

**Authors:** Irantzu Bengoa-Urrengoechea, Sara Malo, María José Rabanaque, María Antonia Sánchez-Calavera, Isabel Aguilar-Palacio

**Affiliations:** 1 Grupo de Investigación en Servicios Sanitarios de Aragón (GRISSA), Fundación Instituto de Investigación Sanitaria de Aragón (IIS Aragón), 50009 Zaragoza, Spain; iranbengoa@gmail.com (I.B.-U.);; 2 Department of Preventive Medicine and Public Health, University of Zaragoza, 50009 Zaragoza, Spain; 3 Network for Research on Chronicity, Primary Care, and Health Promotion (RICAPPS), Instituto de Salud Carlos III, 28029 Madrid, Spain

**Keywords:** diabetes mellitus, type 2, primary health care, COVID-19 pandemic

## Abstract

**Highlights:**

**What are the main findings?**

- Primary care for patients with type 2 diabetes shifted from face-to-face to virtual consultations during the pandemic, resulting in a sustained reduction in face-to-face visits and routine assessments, such as laboratory tests, blood pressure checks, and weight measurements.
- Despite fewer face-to-face contacts and monitoring activities, glycaemic control and other key clinical outcomes (HbA1c, blood pressure and BMI) often remained at levels comparable to those in the pre-pandemic period. However, some vulnerable subgroups experienced deterioration.

**What are the implications of the main findings?**

- The maintenance of disease control in many patients suggests that virtual primary care can support the management of type 2 diabetes during health crises. However, it cannot fully replace face-to-face care, particularly for older adults and socially disadvantaged groups.
- Health systems should develop flexible, patient-tailored models that combine adequately resourced face-to-face care with accessible telemedicine in order to protect chronic disease management and reduce inequalities in future emergencies.

**Abstract:**

**Background:** During the coronavirus disease 2019 (COVID-19) pandemic, changes in healthcare organisation may have affected the management of patients with chronic conditions, such as those with type 2 diabetes (T2D), who require close monitoring. Although there are studies that have analysed the access and management of patients with T2D in primary care (PC) during the pandemic, there is no synthesis of the results that inform us about the care provided and their effect. The aim of this study is to summarise the existing evidence on the care provided in PC settings to patients with T2D during the COVID-19 pandemic, as well as their follow-up care and outcomes, in order to understand the impact of the pandemic on their management. **Methods:** A scoping review was conducted according to the approach described by Arksey and O’Malley. Structured search strategies were developed for each of the selected databases (PubMed, EMBASE and Web of Science). We included articles published in English and Spanish up to 24 March 2026 on access to care for patients with T2D in PC during the pandemic. Two independent reviewers screened titles and abstracts to select studies related to the population, intervention, and outcomes of interest. In cases of disagreement, a third reviewer resolved the discrepancy. One of the reviewers extracted data and summarised them. **Results:** After duplicate removal, 535 records were identified, with 116 articles screened in full text and 26 included. Most studies were conducted in Europe and North America using retrospective designs and electronic health records. During the pandemic period, face-to-face visits and routine screenings generally declined, while telemedicine expanded and may have partially mitigated disruptions in patient contact, although inequalities in access were reported. Disease monitoring and complication screening also declined in several settings. Clinical outcomes such as HbA1c and blood pressure (BP) showed heterogeneous trends, with some studies reporting deterioration and others reporting stability. Some studies also highlighted increased adverse events and greater attention to mental health during care. **Conclusions:** Although telemedicine helped maintain patient follow-up, it could not fully replace face-to-face assessments and was implemented alongside some gaps in clinical monitoring, patient safety, and health equity. These findings underscore the need to maintain structured chronic disease management and to develop safe and inclusive telehealth models.

## 1. Introduction

Diabetes mellitus is a chronic condition characterised by elevated blood glucose levels. According to the International Diabetes Federation, diabetes is one of the fastest-growing global health emergencies of the 21st century. In 2021, it was estimated that 537 million people worldwide had diabetes. This number is projected to reach 643 million by 2030 and 783 million by 2045 [1]. Type 2 diabetes (T2D) accounts for more than 90% of diabetes cases worldwide. It has significant individual and social consequences due to the increased risk of acute and chronic complications, particularly chronic macrovascular and microvascular complications, resulting from abnormal and sustained high blood glucose levels. To prevent the onset of T2D, controlling risk factors and maintaining a healthy lifestyle through diet, physical activity, and smoking cessation are crucial [2]. These goals should be achieved, from a healthcare perspective, mainly through primary care (PC) services.

The COVID-19 pandemic has caused a substantial decrease in access to PC services in some areas, which has had a particularly negative impact on chronic patients who depend on regular healthcare for disease management [3]. This limited access led to deterioration in the control of chronic conditions and increased vulnerability among those affected [4]. Some studies have shown that a reduction in follow-up care has resulted in poorer outcomes and fewer resources for patients with T2D, which has significantly worsened the results of the monitoring indicators, screening and vaccination of patients in PC [5,6], and potentially exacerbated their health issues and increased complications [7].

Understanding the attention received by patients with T2D from PC during the COVID-19 pandemic is crucial for understanding the impact of the pandemic on these patients in terms of control and follow-up. Therefore, the objective of this scoping review is to summarise how people with T2D accessed and used PC during the COVID-19 pandemic and how their disease was monitored and controlled. Our findings will be valuable in suggesting measures to improve the care of these patients in any future comparable scenarios.

## 2. Materials and Methods

### 2.1. Protocol Design

A scoping review was chosen to comprehensively map and characterise the breadth and heterogeneity of the available evidence, rather than to address a narrowly defined question or estimate effect sizes. This approach was particularly appropriate given the diversity of study designs, populations, and outcomes, and allowed us to identify key concepts, research gaps, and areas for future investigation [8] in the field of T2D healthcare attention.

The scoping review was conducted according to the approach described by Arksey and O’Malley (2005) [9]. The approach comprises five steps: (1) Formulation of the research question: identification of the conceptual domain and key constructs relevant to the research question; (2) Identification of relevant studies: systematic mapping of existing literature to these constructs; (3) Selection of eligible studies: extraction and synthesis of data according to each stage of the framework; (4) Collection of data: data extraction from the included studies based on their alignment with the framework’s criteria; (5) Data summary and synthesis of results: integration of findings to address the review’s objectives.

The review protocol has been included in the international platform for the registration of systematic reviews and meta-analysis protocols, INPLASY^®^ (INPLASY registration number: INPLASY202360057) [10], which provides an online database where protocols are maintained as permanent public records and whose website (www.inplasy.com) can be accessed.

### 2.2. Formulation of the Research Question

We used an iterative process, as recommended by Arksey and O’Malley [9], to develop the research question. The main research question aimed to understand how patients with T2D accessed PC services during the pandemic and how this affected their follow-up and disease management.

The following questions guided the review:

- Did patients with T2D continue to attend PC appointments during the COVID-19 pandemic?
- During the COVID-19 pandemic, did general practitioners (GPs) continue monitoring and reviewing patients with T2D?
- Have the results of follow-up care been maintained for patients who received it during the pandemic?
- What are the limitations, gaps, and opportunities of these types of studies?

### 2.3. Identification of Relevant Studies

To identify relevant studies, structured search strategies were developed for each of the selected databases (PubMed, EMBASE, and Web of Science). The choice of databases was driven by their complementary coverage of biomedical and health services research, as well as by their relevance to the population and setting of interest. The strategy in PubMed included the MeSH terms “diabetes mellitus, type 2”, “hypoglycemic agents”, “primary health care”, “primary nursing”, “primary care nursing”, “physicians, primary care”, and “covid-19”. To increase the sensitivity of the search, additional general terms were added to the title and abstract to make the search more sensitive; generic terms such as “non-insulin dependent diabetes mellitus” or “ambulatory care facilities” were added to the title and abstract. This search was adjusted for all other sources of information in accordance with their own specific search criteria. The PRISMA-ScR guidelines were followed [8]. A review protocol was developed and included in the international platform for the registration of systematic reviews and meta-analysis protocols, INPLASY^®^. The search strategies for each of the databases used can be found in Appendix A.

### 2.4. Selection of Eligible Studies

Inclusion criteria

Eligibility criteria were defined a priori on the basis of the review question and the Population–Concept–Context framework. We included observational studies involving patients with T2D that evaluated PC access, healthcare utilisation, continuity of care, follow-up, disease monitoring, clinical management, or relevant clinical outcomes during the COVID-19 pandemic. Eligible studies had to report data collected between 1 January 2020, and 24 March 2026, and be published in English or Spanish. Retrospective cohort, cross-sectional, before-and-after, register-based, and mixed-methods studies were eligible when they provided quantitative findings relevant to the review objectives.

Exclusion criteria

Studies were excluded if they did not include patients with T2D; did not provide PC-specific data; focused exclusively on hospitalised patients, emergency care, specialist care, or higher levels of care; did not assess changes associated with the COVID-19 pandemic; or addressed only the diagnosis or management of COVID-19. We also excluded qualitative studies, grey literature, dissertations, conference proceedings, commentaries, letters, and review articles. Studies including mixed diabetes populations were eligible only when findings for patients with T2D were reported separately or could be meaningfully interpreted in relation to the review question.

The references were processed via the bibliographic manager Mendeley (https://www.mendeley.com, accessed on 25 March 2026) and later exported to Rayyan software (https://www.rayyan.ai/, accessed on 25 March 2026) for data management and duplicate removal. Articles were included if they met the inclusion criteria and excluded if they did not. Two independent reviewers (I.B.-U. and S.M.) screened the titles and abstracts to select studies related to the population, intervention, and outcomes. In cases of disagreement, a third reviewer (I.A.-P.) resolved the discrepancy.

The full texts were then assessed against the same pre-specified criteria. Reasons for exclusion at the full-text stage were recorded and are presented in the PRISMA flow diagram. The screening process was not based on a quality assessment threshold, because the objective of this scoping review was to map the available evidence rather than to estimate a pooled effect or exclude studies according to risk of bias. Nevertheless, the relevance of each article to the population, PC setting, pandemic period, and outcomes of interest was assessed systematically.

### 2.5. Collection of Data

During this screening phase, we searched for studies whose full texts potentially met the inclusion criteria. Any full-text studies that did not meet the inclusion criteria were excluded, and the reasons for exclusion were reported. After identifying the papers that met the defined criteria, we thoroughly read and classified them on the basis of their characteristics and relevant information. The final search results are presented in a PRISMA flowchart.

Data extraction was performed using a standardised charting form developed a priori and piloted on a sample of included studies. For each article, one of the reviewers extracted the following information: author, year, title, journal, country, study design, sources of information, study duration, population characteristics, indicators measured, main results, conclusions, and limitations. Data extraction and summarisation were carried out by a single reviewer (I.B-U.), and any uncertainties were discussed with the other two reviewers (I.A-P and S.M). This process allowed us to draw robust conclusions about the care of patients with T2D in primary care during the COVID-19 pandemic.

### 2.6. Data Summary and Synthesis of Results

First, all of the information extracted from each article was presented in tables to facilitate a comparison of the general characteristics and outcomes of the included studies. We then conducted a descriptive and narrative synthesis of the findings. Through an iterative process, the quantitative outcomes (such as PC visits, follow-up activities, vital sign recording, laboratory measurements, and referrals) were grouped into two overarching domains: access to healthcare services and disease management. Within each domain, we summarised the direction of change (increase, decrease, or no relevant changes) and highlighted consistencies and divergences across countries, health systems, and patient subgroups. We also examined whether differences in findings were plausibly related to contextual and methodological factors, including the organisation and pre-pandemic digital maturity of PC, the intensity and timing of pandemic restrictions, patient characteristics, access to home-monitoring resources, outcome definitions, and follow-up duration. These factors were used to structure the interpretation of heterogeneity and were not considered evidence of causal relationships. After being classified and summarised, the results were interpreted and discussed in light of the existing literature without calculating the pooled estimates or inferring causal effects given the heterogeneity of the data.

### 2.7. Patient and Public Involvement

Patients and the public were not involved in the design or completion of this study.

## 3. Results

**Figure 1:**
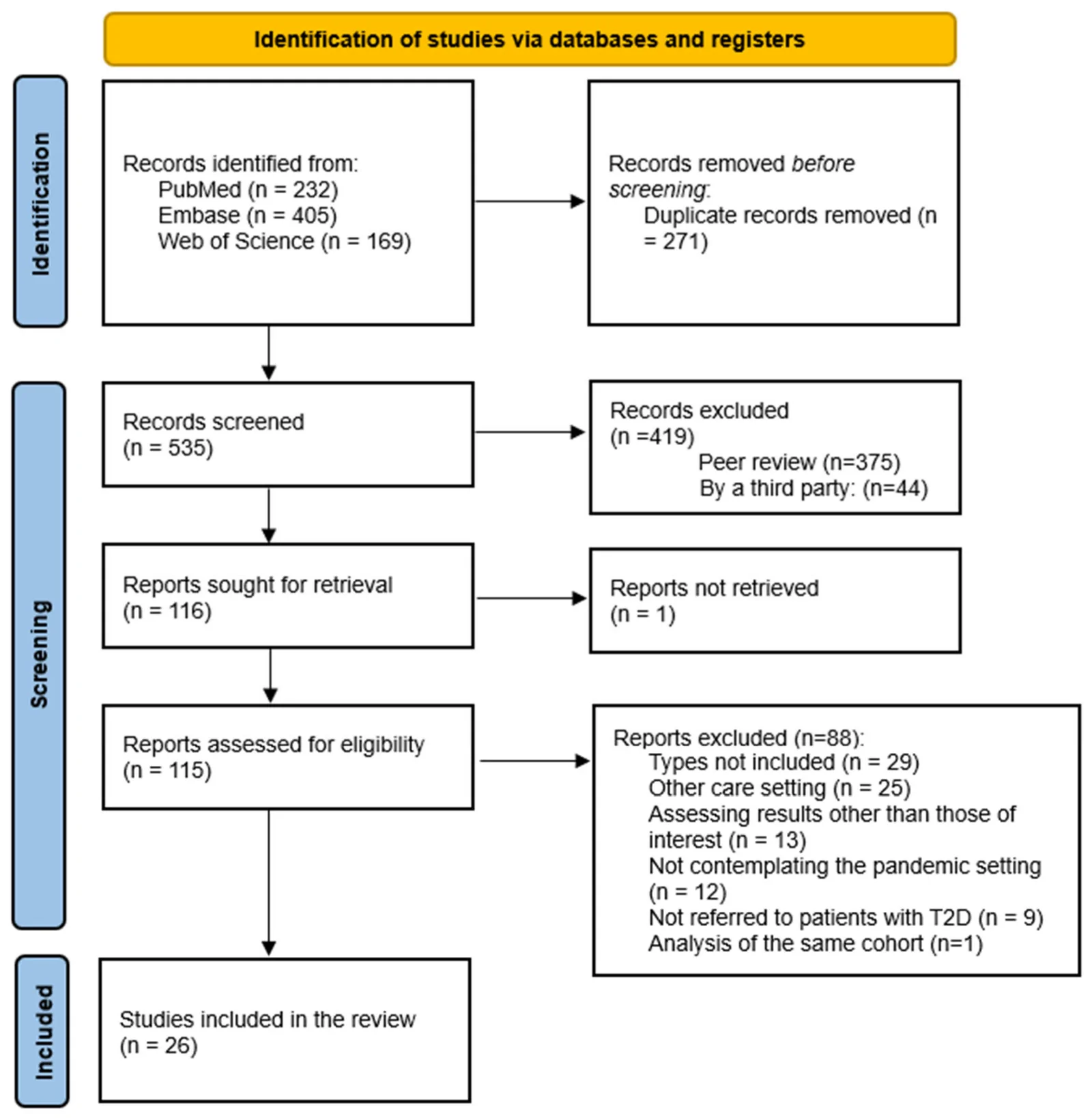
PRISMA 2020 flow diagram for new systematic reviews, which included searches of databases and registers only, From: [11].

After removing duplicates, 535 articles were identified for review selection. Following the review of titles and abstracts, 116 articles met the inclusion criteria. Once we had access to the full-text articles, we read and screened them and found that 26 focused on the care of patients with T2D in PC during the pandemic (Figure 1).

The main characteristics of the studies are summarised in Table 1. 

Europe had the highest number of publications (57.7%). The United States and Canada were the only countries from the Americas included in the review, accounting for 34.6% of the publications. Three studies represented Asia, and none from Africa. Most of the studies (77.9%) were retrospective cohort studies, followed by retrospective cross-sectional studies. The most commonly used source of data was electronic health records. In most cases, data were extracted for two time periods to compare indicators before and during the pandemic, or for three time periods to compare indicators before, during, and after the pandemic.

The included studies exhibited significant heterogeneity in terms of population size, ranging from small cohorts to large population-based analyses. The smallest sample sizes were reported by Freeman et al. [37] and van Grondelle et al. [29]. Freeman et al. studied 159 adults with T2D (52 in the pre-pandemic cohort and 141 during the pandemic), while van Grondelle et al. surveyed 171 participants (alongside collecting routine care data from 607 individuals). Other relatively small studies included those by Cuevas Fernández et al. [15], which involved 587 participants, and by Koh et al. [24], which involved 644 participants. In contrast, Moin et al. [21] reported the largest cohorts by far, with 1,393,404 individuals in the pandemic cohort and 1,415,490 and 1,444,000 in the two pre-pandemic cohorts. Similarly, Carr et al. [14] analysed data from 618,161 adults with T2D in the United Kingdom, and Coma et al. [12] included over 300,000 individuals in Spain.

After the results of the 26 articles were analysed, two topics of interest were identified: access to healthcare services and results regarding disease management. However, the heterogeneity of outcome measures, sample sizes, and study designs limited our ability to present quantitative indicators of change in a concise, directly comparable format. Within each domain, we summarise the main outcomes assessed and the direction of change. We also present a table showing the sample size, outcome categories, and direction of effect (increase, decrease or no significant change) to highlight recurring patterns across studies. Readers seeking additional detail are referred to the Supplementary Materials. Regarding the collection of outcomes measured in the studies, follow-up activities such as recording vital signs, screening for retinopathy and diabetic foot, during visits were the most common, followed by laboratory measures. Table 2 shows how many studies address each of the listed outcomes.

### 3.1. Access to Health Services

The COVID-19 pandemic caused a massive global disruption in physical access to PC. In Ontario, Canada, the probability of a face-to-face visit to a GP decreased by 47% during the first six months of the pandemic [28]. Similarly, researchers in the United Kingdom observed that health checks were reduced by 76–88% in April 2020 compared to historical trends [14]. In Germany, new diagnoses of T2DM initially dropped by 35.7% as patients avoided practices [25]. In Denmark, while the total number of contacts per patient actually increased from 6.3 to 8.3 annually, the nature of these visits shifted significantly toward administrative staff and virtual modalities [31].

To compensate for the lack of face-to-face care, health systems implemented a rapid transition to virtual care. In Qatar, 60.6% of all consultations during the pandemic were virtual [20]. In Ontario, the use of virtual visits increased by over 330% [23]. In Singapore and Ontario, telephone consultations were shown to be non-inferior to face-to-face visits for maintaining glycaemic control in patients who were already relatively stable (HbA1c < 9%) [23,24]. In Finland, the impact on continuity was cushioned by a system that had already relied heavily on telemedicine before the pandemic, allowing even patients over 70 to maintain contact [18]. This contrast suggests that the effect of telehealth depended not only on its availability during the pandemic, but also on its prior integration into PC workflows and on the capacity of services and patients to use it effectively.

However, this transition revealed significant social and systemic disparities [29]. In the United States, patients identifying as racial or ethnic minorities, or those with Medicaid or no insurance, were significantly more likely to miss routine screenings [17]. The digital divide, along with limited technological literacy, was also identified as a significant barrier to the effective implementation of telehealth platforms [23]. In the Netherlands, patients enrolled in structured disease management showed higher resilience, experiencing a less pronounced decrease in outpatient service utilisation compared to those not enrolled. Furthermore, the reduction in routine PC services led to long-term pressure on the system. In the Netherlands, downsizing chronic disease management programmes in 2020 was associated with an 11% increase in hospitalisations and a rise in regular GP visits in 2021 [32].

### 3.2. Disease Management

The intensity of clinical monitoring declined significantly during the pandemic, with routine screenings for diabetic complications being among the areas most affected. In Catalonia, PC practices with limited face-to-face capacity saw a 60.7% decrease in diabetic foot screenings and a significant drop in retinopathy exams [15]. Similar trends were noted in Canada and the Netherlands, where BP and HbA1c monitoring frequency fell sharply [29,37]. Laboratory testing was also impacted. In Switzerland, 19% and 14% of HbA1c and BP records were missing, respectively, due to the pandemic [16].

The impact on clinical outcomes (HbA1c and BP control) was heterogeneous across the studied populations. In Hong Kong and Qatar, no clinically relevant deterioration in mean HbA1c or BP levels was found after one year [20,34]. Conversely, data from Northern California and the Netherlands showed significant increases in mean HbA1c and systolic BP [26,29,35]. In the Minnesota health system, the proportion of patients meeting the optimal HbA1c guidelines (<8%) fell from 73.1% to 58.2% [17]. Interestingly, some patients with very poor control prior to the pandemic (very high baseline HbA1c) showed significant improvements during lockdown [35], likely due to the medical prioritisation of high-risk cases or improved self-care routines while staying at home [34]. These contrasting findings indicate that short-term clinical stability did not necessarily imply preserved monitoring, as patients with stable disease or continued engagement with healthcare services may have been more likely to have the outcomes recorded. Differences in baseline disease control, patient selection, follow-up duration, and the organisation of care may therefore have contributed to the variability observed across studies.

Patient safety and mental health emerged as critical secondary management concerns. A study in Texas indicated that the conversion to majority telehealth marginally increased adverse drug events (ADEs), such as insulin-associated hypoglycaemia, in high-risk populations [36]. On a positive note, some Canadian clinics reported a significant increase in depression screening during diabetes appointments (from 47.1% to 73.9%) [37], reflecting an adaptive response to the pandemic’s psychosocial toll. Patients reported that facilitators for success during this period included strong social support from family, religious faith, and the use of personal medical devices for home monitoring [29].

Across the two domains, the findings showed a consistent separation between service contact, clinical monitoring, and clinical outcomes. PC contacts were often maintained through the rapid expansion of virtual consultations, whereas face-to-face assessments, laboratory testing, and complication screening generally declined. However, this reduction in monitoring did not uniformly translate into worse short-term HbA1c, BP, or BMI outcomes. This pattern suggests that the preservation of clinical outcomes and the preservation of comprehensive monitoring were not equivalent processes. The apparent stability of some clinical indicators may partly reflect the inclusion of patients who remained engaged with care or had relatively stable disease before the pandemic, whereas patients who disengaged from services and those with unmet monitoring needs may be underrepresented in the available data. Differences between studies should therefore be interpreted in relation to both the care model and the population that remained observable within each healthcare system.

## 4. Discussion

This scoping review identified 26 studies showing that access to PC for people with T2D declined during the COVID 19 pandemic, with fewer follow-up visits and, in some cases, deterioration in clinical outcomes. In line with previous research, the pandemic exacerbated pre-existing disparities in healthcare access, with individuals living in low-income areas and older adults emerging as particularly vulnerable groups. Reductions in PC and hospital visits were attributed not only to organisational and logistical barriers but also to the patients’ fear of acquiring SARS-CoV-2 infection in healthcare settings [17,19,22,33,35,37].

Across the included studies, people with T2D experienced disruptions in continuity of care and medication adherence, which likely contributed to widening health inequalities during the pandemic. Several studies reported a decrease in GP visits, consultations, and prescription rates for chronic medications, along with changes in prescribing patterns. Difficulties in adherence were particularly evident among patients requiring specialist input or new prescriptions, underscoring the impact of structural barriers to accessing both primary and specialist care [13,18,19,27,29,32,34].

The rapid expansion of telemedicine partially mitigated the reduction in face-to-face encounters but also introduced new challenges, especially for socially and digitally disadvantaged groups. Limited Internet connectivity, difficulties in using electronic devices and lower levels of digital literacy were consistently highlighted as barriers and reinforced existing inequities in access to care. Some studies showed that women, Black patients, and those with public insurance coverage (e.g., Medicare/Medicaid) were more likely to face delays in scheduling appointments or to express reluctance to return to healthcare facilities. These findings contrast with other reports, which described higher consultation rates in older adults and women and better follow-up indicators in these groups, suggesting that the impact of the pandemic was highly context dependent and mediated by the local organisation of care and social determinants [13,17,22,26,27,32,37].

Although virtual consultations appeared to be effective in maintaining follow-up for a substantial proportion of patients, important limitations were identified. These included technical problems, the inability to perform physical examinations and challenges in assessing complex clinical situations. Virtual visits may be particularly suitable for younger working adults, administrative issues, or monitoring stable chronic conditions previously evaluated in person; however, they cannot fully replace regular face-to-face care. This is especially relevant for older individuals, patients with sensory or cognitive limitations, and those with new symptoms or complications requiring physical examination [17,19,21,22,23,24,27,37].

Several strategies were implemented to preserve continuity of care for chronic patients, including the involvement of community pharmacists, home delivery or home based medication programmes, and simplified prescription procedures. These interventions helped to mitigate care disruptions while complying with infection control measures. Nonetheless, a sizeable proportion of patients reported delaying or avoiding care, citing provider initiated cancellations, safety concerns and financial or insurance related problems. These findings underline the importance of developing accessible, patient centred models of care that are resilient to system shocks and capable of maintaining essential follow-up for vulnerable populations [34].

Some of the included studies described a reduction in the incidence of newly diagnosed T2D during the pandemic period, together with slightly higher HbA1c levels at diagnosis. This pattern is consistent with under diagnosis and diagnostic delay, likely due to the reduced utilisation of PC services and screening activities. In addition, poorer intermediate outcomes (higher HbA1c, BP or BMI) were reported in certain cohorts. Possible explanations include the avoidance of healthcare facilities due to infection fears, reduced opportunities for structured lifestyle counselling, and the convenience of avoiding travel to health centres. In some settings, patients with uncontrolled diabetes (e.g., HbA1c > 9%) were more likely to attend virtual visits, but these contacts may have been less effective than in person consultations in improving glycaemic control. Face-to-face visits with GPs and nurses may contribute to a greater awareness of clinical parameters and reinforce the importance of lifestyle modification, which may be more difficult to achieve through remote care alone [12,18,25,27,30,35,38]. Taken together, the findings indicate that the pandemic affected different dimensions of diabetes care in different ways. Maintaining contact through virtual consultations did not necessarily preserve the completeness of clinical monitoring, while reduced monitoring did not invariably result in immediate deterioration in HbA1c, BP, or BMI. The overall effect therefore appears to have been shaped by the interaction between the care modality, baseline disease control, patient ability to self-manage, and the capacity of PC services to provide complementary face-to-face assessments. This interpretation helps reconcile the apparently divergent findings across studies without assuming that the observed associations were causal.

In a considerable number of studies, no significant differences in HbA1c, BP, or BMI were observed when comparing the pre-pandemic and pandemic periods. The strongest predictor of maintaining guideline recommended HbA1c levels during the pandemic was having achieved those targets beforehand. Conversely, worse outcomes were observed in individuals with previously poor glycaemic control, while in other analyses, increases in HbA1c and other risk factors were more pronounced among lower risk patients (younger individuals with previously controlled HbA1c). A small but statistically significant inverse association between the number of face-to-face visits and HbA1c was reported, which may reflect reverse causality, as more engaged or health conscious patients are also more likely to request consultations [12,13,14,16,32,34,38]. These findings should nevertheless be interpreted cautiously, because the patients for whom clinical outcomes were available may differ systematically from those who experienced prolonged disengagement from care or were not reached during the pandemic.

### 4.1. Contextual Explanations for Cross-Country Variation

The differences observed across countries and healthcare systems are likely to reflect the interaction of several contextual factors rather than a single characteristic of the healthcare model. First, telemedicine infrastructure and previous experience with remote care appear to have influenced the ability of PC services to maintain contact. Finland provides an example of a setting in which pre-pandemic reliance on telemedicine may have facilitated continuity, whereas the rapid implementation of virtual care in other settings was accompanied by technical barriers, differences in digital literacy, and unequal access to devices or connectivity.

Second, the organisation and financing of PC may have affected how patients accessed services during the pandemic. Insurance status and financial barriers were associated with missed or delayed care in some populations, while differences in local service organisation influenced the balance between administrative, virtual, and face-to-face contacts. These findings should not be interpreted as evidence that one financing model was uniformly superior, because the available studies examined different populations, outcomes, and healthcare settings. Rather, they suggest that the practical consequences of financing arrangements depended on how rapidly services could be reorganised and whether patients had alternative routes to obtain care.

Third, pre-existing chronic disease management programmes may have provided organisational resilience. In the Netherlands, patients enrolled in structured disease-management programmes experienced a less pronounced reduction in outpatient utilisation, whereas downsizing such programmes was followed by increased hospitalisations and regular GP visits in the subsequent period. This suggests that continuity mechanisms established before the pandemic may have helped preserve follow-up, although the observational nature of the evidence prevents a definitive causal interpretation.

Finally, the timing and intensity of pandemic restrictions, together with local decisions regarding the suspension of routine care, influenced the opportunity to perform face-to-face examinations, laboratory testing, and complication screening. The marked reduction in diabetic foot and retinopathy screening in Catalonia, the decrease in monitoring in Canada and the Netherlands, and the missing HbA1c and BP records reported in Switzerland illustrate how service reorganisation affected monitoring differently across settings. These contextual differences may help explain why some studies reported relatively stable short-term clinical outcomes while others observed deterioration in glycaemic control or BP.

Patient empowerment and previous self-care training may have contributed to the maintenance of disease control when professional supervision was reduced. However, the included evidence does not establish that greater autonomy caused the observed maintenance or improvement in glycaemic outcomes [15]. In one study, patients reported changes in self-monitoring and self-management behaviours, including dietary and physical-activity adjustments while another study identified personal medical devices and social support as facilitators of diabetes management during the pandemic [30]. These findings suggest that self-management resources may have supported some patients during service disruption, but their contribution cannot be separated from other factors, such as baseline disease control, access to medication, social support, and continued contact with healthcare services [29].

Conversely, some studies suggested that patients who relied more heavily on regular professional monitoring and structured clinical reminders may have been particularly affected by service disruptions [12,32]. In the Netherlands, the downsizing of structured chronic disease management programmes in 2020 was followed by an increase in hospitalisations and regular PC visits in 2021. Although this temporal pattern is compatible with a possible disruption of ongoing disease management, the observational design does not allow us to determine whether reduced professional follow-up caused subsequent deterioration or whether other changes in patient characteristics, healthcare utilisation, or system organisation contributed to these findings [32]. 

Certain population groups may require proactive healthcare support, particularly when access to routine care is disrupted. In the included studies, individuals with lower socioeconomic status or limited insurance coverage were less likely to achieve optimal control targets or receive routine monitoring during the pandemic. These findings are consistent with the possibility that structural inequities affected access to care and disease-management resources, although the available observational data cannot establish the specific mechanisms underlying these differences [17].

Taken together, these findings suggest that dependence on regular healthcare contact and social vulnerability may be relevant markers of increased risk during major disruptions to PC. They also support the potential value of targeted strategies that combine accessible professional follow-up with support for self-management, particularly among patients facing socioeconomic, clinical, or digital barriers [17,32]. However, the included studies do not allow us to determine whether strengthening self-management would independently improve outcomes, and such strategies should therefore be evaluated in prospective or appropriately designed comparative studies. 

### 4.2. Strengths and Limitations of This Review

This scoping review followed a predefined and transparent search and synthesis strategy; however, several limitations must be acknowledged. The restriction to English- and Spanish-language publications may have introduced language bias by excluding relevant evidence published in other languages, particularly evidence from regions where the organisation of PC and the implementation of telehealth may differ substantially from those represented in our review. Consequently, the findings may overrepresent evidence from English- and Spanish-speaking settings and may not fully capture the international diversity of healthcare-system responses during the pandemic. Although both MeSH terms and free-text words were used, the application of a defined search strategy and strict eligibility criteria might have excluded studies addressing similar questions in different formats. 

The exclusion of grey literature, including governmental and institutional reports, technical evaluations, preprints, conference proceedings, and other non-peer-reviewed sources, may also have limited the identification of rapidly emerging evidence. This is particularly relevant to telehealth, as many interventions were implemented and evaluated during the pandemic before they were formally published in indexed journals. These sources may have provided valuable information on early implementation processes, service adaptations, barriers to access, digital inequalities, and healthcare-system responses that are not fully captured in the peer-reviewed literature.

Accordingly, the results should be interpreted as a synthesis of the peer-reviewed evidence available in English and Spanish rather than as an exhaustive account of all pandemic-related experiences. Although these restrictions improved the feasibility and consistency of the review, they may have contributed to publication and language bias and may have resulted in the underrepresentation of negative, unsuccessful, or context-specific telehealth experiences. Future reviews should consider multilingual searches and the systematic inclusion of grey literature to provide a more comprehensive assessment of telehealth implementation and healthcare-system adaptation during health emergencies. Some primary studies reported concerns about representativeness due to small sample sizes for certain minority ethnic groups or the over-representation of specific regions or healthcare systems. Furthermore, the restrictions implemented by official authorities varied considerably between countries, and patient and PC responses are highly dependent on the organisation of healthcare systems (including payment models), which also differ substantially across settings. 

The analysed studies could be considered primarily limited by its observational and retrospective nature. This prevents definitive causal relationships from being established between pandemic-related restrictions and the observed clinical outcomes [12,17,21,32,34,36]. This limitation is particularly relevant when interpreting apparent improvements in glycaemic control or reported self-management behaviours, because the included studies generally could not establish temporal or causal relationships between these factors and clinical outcomes. Furthermore, several studies may have limited generalisability as they were conducted within specific geographical areas, single healthcare systems, which may not reflect national or global trends [3,19,22,24,27,28,31,33]. A significant challenge identified in a considerable number of the articles was the high incidence of missing clinical data in electronic health records, particularly with regard to laboratory results such as HbA1c and physical measurements such as BMI and BP. This likely introduced a selection or information bias by focusing only on patients who actively sought care [13,15,16,19,25,26,29,36,37]. Furthermore, some studies acknowledge difficulties in evaluating the quality of virtual care and in distinguishing between various consultation methods [12,18,20]. Finally, the limited follow-up duration of many studies means that the long-term impact and potential ‘rebound effects’ of delayed screenings and routine care for chronic complications in the years following the peak of the pandemic remain unclear [13,19,21].

Another important limitation concerns the handling of heterogeneous populations. The included studies differed markedly in sample size, demographic composition, socioeconomic and ethnic characteristics, health-system organization, and pandemic phase. In addition, two studies did not differentiate between type 1 and T2D, and we included their findings under the assumption that given the higher proportion of patients with T2D with respect to type 1, the results predominantly reflect patients with T2D. This heterogeneity, together with the inclusion of mixed diabetes populations in some studies, limits the comparability of results across settings and may affect the generalizability of the findings. The narrative synthesis approach adopted in this scoping review was intended to accommodate this diversity, but it precludes formal subgroup analyses or pooled estimates.

Synthesising results from heterogeneous populations, health systems, and pandemic phases is inherently challenging, and causal inferences cannot be drawn from the aggregated evidence. Importantly, the studies included in this review could not capture outcomes in individuals who did not access the healthcare system during the pandemic due to restrictions, system overload, or personal barriers. These “invisible” patients likely include those at highest risk of complications and may have experienced the worst outcomes. Finally, the impact of reduced monitoring and visit frequency on patient-reported outcomes, such as quality of life, psychological well-being, and perceived continuity of care, was rarely evaluated and could not be analysed in this review.

Finally, the exclusion of qualitative studies limits the review’s ability to capture patient experiences, barriers to telehealth, and perceptions of continuity of care, which are highly relevant to understanding healthcare utilization during the pandemic. Future syntheses that integrate qualitative evidence alongside quantitative findings would provide a more comprehensive understanding of the mechanisms underlying the observed changes in access and disease management.

Despite these limitations, this review also has notable strengths. A comprehensive search across multiple databases was conducted, and a broad range of study designs and settings was included, which enhances the external validity and richness of the findings. In addition, it is broad in scope because it includes a variety of study designs. The synthesis provides a useful framework for future research, clinical practice and health-policy planning aimed at strengthening PC-based diabetes management and improving preparedness for future crises.

## 5. Conclusions

The COVID-19 pandemic was associated with widespread disruption to PC, including a shift from face-to-face consultations towards virtual consultations and a reduction in routine clinical monitoring. Across the included studies, fewer laboratory tests, physical assessments of BP and weight, and screening examinations for retinopathy and diabetic foot problems were reported during the pandemic period.

These findings suggest that although telemedicine was a useful tool for maintaining contact, it could not fully replace the physical assessments required for comprehensive T2D management. Although many patients maintained stable glycaemic control, some vulnerable subgroups experienced poorer outcomes during the pandemic period. These heterogeneous findings occurred alongside reduced face-to-face care, but the observational evidence does not establish that reductions in face-to-face monitoring caused clinical deterioration. The accelerated implementation of digital health may have interacted with pre-existing inequalities, including a digital divide affecting some older adults and socioeconomically disadvantaged groups. The findings support the development of hybrid and flexible care models combining face-to-face care with inclusive telemedicine to promote structured chronic disease management, health equity, and patient safety during future health crises.

## Figures and Tables

**Table 1:** Study characteristics.

Author (Year)	Country	Study Design	Study Period	Study Population	Healthcare Settings
Coma et al. (2021) [12]	Spain	Retrospective cohort study	2019–2020; pre-pandemic vs. pandemic (≈24 months, consecutive)	n = >300,000 adults with T2D, aged >14 years	287 PC practices
Kowall et al. (2021) [13]	Germany	Retrospective cohort study (longitudinal before-after comparison)	2018–2020; pre-pandemic vs. post-lockdown (3 × 6-month windows, ≈18 months total analysed)	n = 32,399 adults with T2D, mean age 68 years, 54% male	837 GPs and internists
Carr et al. (2022) [14]	United Kingdom	Retrospective cohort study (open-cohort, time-series design)	2010–2020; pre-pandemic baseline vs. March–December 2020 pandemic period (baseline ≈ 122 months + pandemic ≈ 10 months)	n = 618,161 adults with T2D, median age 68 years, 44% female	1744 general practices
Cuevas Fernández et al. (2022) [15]	Spain	Multicentric retrospective observational study	2019–2021; pre-pandemic vs. pandemic vs. recovery (≈36 months, consecutive)	n = 587 adults with T2D at baseline, mean age 66 years, 54% male (558 alive at follow-up)	1 PC centre; 12 medical/nursing slots
Di Gangi et al. (2022) [16]	Switzerland	Retrospective cohort study	2018–2021; pre-pandemic vs. pandemic (2 × 12-month baseline cohorts + ≈12-month follow-up, ≈36 months total)	n = 27,043 adults with diabetes (type 1 and type 2), aged ≥18 years, ~43% female	191 GPs practices
Hooker et al. (2022) [17]	USA	Retrospective cohort study	2019–2020; pre-pandemic vs. pandemic (14.5 + 9.5 ≈24 months)	n = 42,942 adults with T2D, mean age 62.2 years, 53.3% male	55 PC clinics
Inglin et al. (2022) [18]	Finland	Retrospective cohort study	2019–2020; pre-pandemic vs. lockdown vs. post-lockdown (three periods per year; ≈6 months pre + ≈5.5 months lockdown + ≈7 months post each year)	n = 11,458 adults with T2D, mean age 69.4 years, 45.6% women	All primary healthcare and specialised healthcare services
Jug et al. (2022) [19]	Croatia	Retrospective longitudinal observational study	2018–2020; pre-COVID vs. COVID (2 × 12-month pre-COVID years + 12-month COVID year, ≈36 months total)	n = 648 adults with T2D, diagnosed before 2018, without SARS-CoV-2 infection	8 family medicine practices
Khmour et al. (2022) [20]	Qatar	Cross-sectional retrospective data analysis	2019–2020; pre-pandemic vs. early pandemic (3-month pre-pandemic window + 6-month pandemic window, ≈9 months total)	n = 17,413 adults with T2D, mean age 49.8 years, 56.6% male	27 health centres
Moin et al. (2022) [21]	Canada	Before-and-after observational study	2019–2020; pre-pandemic vs. early pandemic (2 × 6-month pre-pandemic cohorts + 6-month pandemic cohort, ≈18 months total)	n = 1,393,404 adults with diabetes (non-gestational) in the pandemic cohort; two pre-pandemic cohorts: n = 1,415,490 and n = 1,444,000	PC, specialist, emergency department, and hospital settings
Strumann et al. (2022) [22]	Germany	Retrospective cohort study	2016–2022; pre-pandemic vs. pandemic (≈48 months pre-COVID + ≈24 months during COVID, ≈72 months total, quarterly data)	n = 4182 adults with T2D (13.6% of 30,734 primary care patients)	9 private PC practices
Cheng et al. (2023) [23]	Canada	Retrospective cross-sectional study	2018–2021; pre-pandemic vs. pandemic (24-month pre-pandemic period + 12-month pandemic period, ≈36 months total)	n = 16,845 adults with T2D with ≥1 healthcare contact	PC practices
Koh et al. (2023) [24]	Singapore	Retrospective propensity score matched cohort study	2020; pandemic lockdown period (≈5.5–7.5 months continuous lockdown)	n = 644 adults with T2D, HbA1c ≥7.0%, aged 21–80 years	1 PC polyclinic
Kowall et al. (2023) [25]	Germany	Retrospective cohort study (before-after comparison)	2018–2021; pre-pandemic vs. pandemic (≈20–21 months pre-pandemic + ≈21 months pandemic, ≈41–42 months total)	n = 21,747 adults with newly diagnosed T2D (pre-pandemic) and n = 20,513 (pandemic)	970 GPs and specialists including diabetologists
van den Berg et al. (2023) [26]	Netherlands	Retrospective cohort study	2019–2020; pre-pandemic vs. pandemic (10-month 2019 period vs. 10-month 2020 period; sensitivity: 4-month windows, ≈20 months analysed)	n = 182,048 adults with T2D at baseline, 47% female, mean age 69.0 years	Not specified
Cuevas Fernández et al. (2024) [27]	Spain	Multicentric retrospective follow-up study	2019–2021; pre-pandemic vs. pandemic (≈36 months total, consecutive)	n = 3543 adults with T2D, 50% women, 38% aged ≤65 years	7 PC health centres
Senthinathan et al. (2024) [28]	Canada	Retrospective cohort study	2019–2021; pre-pandemic vs. early pandemic vs. later pandemic (3 × 6-month March–September windows, ≈18 months total analysed)	n = 39,401 adults with T2D, aged ≥18 years	280 family physicians
van Grondelle et al. (2024) [29]	Netherlands	Mixed-methods prospective observational cohort study (survey, semi-structured interviews, and routine care data analysis)	2019–2022; pre-pandemic vs. pandemic (one pre-pandemic year vs. first pandemic year, within a broader September 2020–January 2022 frame; ≈24 months around the comparison)	Survey: n = 171 adults with T2D; routine-care data: n = 607 adults with T2D; interviews: n = 10 adults with T2D from ethnic minorities	1 PC centre
Freeman et al. (2025) [30]	Canada	Two-phase mixed-methods study: (i) retrospective pre-post cohort study (quantitative); (ii) qualitative descriptive study using semi-structured interviews	2018–2021; pre-pandemic vs. pandemic (21-month pre-pandemic period + 21-month pandemic period, ≈42 months total)	n = 84,617 adults with diabetes aged ≥50 years, mean age 68.2 years, 47.3% female	13 practice-based research networks
Nielsen & Andersen (2025) [31]	Denmark	Register study/semi-longitudinal cohort study	2019–2021; pre-pandemic vs. pandemic (12-month pre-pandemic period + 12-month pandemic period, ≈24 months total, consecutive)	n = 48,650 enlisted patients before and n = 47,207 during the pandemic (general primary care population; T2D analysed as a subgroup)	11 GP clinics
Rijpkema et al. (2025) [32]	Netherlands	Retrospective observational study	2019–2021; pre-pandemic vs. pandemic (three consecutive years with quarterly analysis, ≈36 months total)	n = 15,247 adults with T2D in chronic disease management programmes, aged ≥18 years	~500 GP practices
Weaver et al. (2025) [33]	Canada	Retrospective matched cohort study	2018–2022; pre-COVID-19 vs. COVID-19 (≈24 months pre + ≈24 months during, ≈48 months total)	n = 28,101 adults with diabetes (14,107 First Nations, 13,994 non-First Nations), aged ≥18 years	All GP visits, emergency departments, and hospital facilities
Wong et al. (2025) [34]	China	Retrospective cross-sectional study	2019–2021; pre-pandemic vs. post-pandemic (two 1-month recruitment windows in consecutive years, plus comparison of 2019 vs. 2020 cohorts; ≈2 months of recruitment, with yearly cohorts behind)	n = 3600 adults with T2D (1800 per group) with regular follow-up and HbA1c measured	8 public PC clinics
Yamamoto et al. (2025) [35]	USA	Retrospective cohort study	2019–2020; pre-SiP vs. post-SiP (7-month pre-SiP window + 7-month post-SiP window, ≈14 months total, separated by SiP)	n = 168,621 adults with diabetes (type 1 and type 2), aged ≥18 years, ≥1 HbA1c in both periods	Not reported as individual PC practices
Young et al. (2025) [36]	USA	Retrospective observational study	2019–2020; pre-COVID vs. slowdown vs. recovery (9-month pre-COVID period + 3-month slowdown + 7-month recovery, ≈19 months total)	n ≈ 8000 adults with T2D on high-risk regimens (insulin or sulfonylureas), aged ≥18 years	1 PC centre
Freeman et al. (2026) [37]	Canada	Retrospective pre-post cohort pilot study	2019–2021; pre-pandemic vs. pandemic (30-day pre-pandemic index window with ±2-month extraction + 12-month pandemic period, ≈14–15 months total)	n = 159 adults with T2D; pre-period cohort n = 52, during-period cohort n = 141; mean age in pre-period 62.1 years	Not specified

Primary care (PC); general practitioners (GPs); type 2 diabetes (T2D); glycated haemoglobin (HbA1c).

**Table 2:** Outcomes measured.

Outcome	↑ Increased	↓ Decreased	↔ No Change	N Studies Reporting	Studies Reporting
Access to Healthcare					
Total primary care visits	3	15	1	19	[14,15,16,17,18,19,20,21,22,23,25,26,27,28,29,31,32,33,36]
Face-to-face visits	2	16	2	20	[12,14,15,17,18,19,20,21,23,24,26,27,28,29,30,31,32,33,36,37]
Virtual consultation	17	0	0	17	[14,17,18,19,20,21,23,24,26,27,28,29,30,31,33,36,37]
Continuity of care	0	8	9	17	[12,15,16,18,19,21,22,23,24,26,29,30,31,32,34,36,37]
Emergency assistance	1	3	2	6	[18,21,25,32,33,36]
Specialist access	2	4	0	6	[19,21,25,30,32,36]
Care for complications	1	1	1	3	[21,32,37]
Referrals	1	2	1	4	[19,22,23,25]
**Disease management**					
T2D diagnosis	0	2	2	4	[12,25,31]
Follow-up	0	10	0	10	[15,16,17,18,19,25,26,29,30,32]
Requested controls	0	8	0	8	[14,15,16,21,23,25,26,29]
HbA1c request	0	14	2	16	[12,14,15,16,17,18,19,21,23,25,26,27,28,29,30,36,37]
HbA1c result	5	3	11	19	[12,13,15,16,17,19,20,22,23,24,25,26,27,28,29,30,34,35,37]
BP measurement	1	10	1	12	[12,14,15,16,23,25,26,28,29,30,37]
BP result	5	3	7	15	[12,13,15,16,19,22,23,25,26,28,29,30,34,35,37]
Weight/BMI measurement	0	8	1	9	[14,15,16,19,23,26,29,34,37]
Weight/BMI result	1	2	9	13	[13,15,16,19,20,22,23,25,26,29,34,35,37]
Laboratory determinations	0	10	1	11	[14,15,16,18,19,21,23,26,29,30,37]
Laboratory results	4	2	3	9	[13,15,16,19,23,25,26,29,37]
Eye examination	0	4	1	6	[12,14,15,19,21,37]
Foot examination	0	1	1	4	[12,14,21,37]
Controlled patients	0	7	7	14	[13,15,16,17,20,22,23,25,26,27,29,30,34,35]
Prescriptions	4	1	4	9	[14,15,16,21,25,30,33,35,36]

Type 2 diabetes (T2D); glycated haemoglobin (HbA1c); blood pressure (BP); body mass index (BMI).

## Data Availability

No new data were created or analyzed in this study.

## Supplementary Materials

The following supporting information can be downloaded at: https://www.mdpi.com/article/10.3390/healthcare14172804/s1, Table S1. Access to healthcare—direction of change; Table S2. Disease management—direction of change.

## Abbreviations

The following abbreviations are used in this manuscript:

T2D	Type 2 diabetes
PC	Primary care
GP	General practitioner
HbA1c	Glycated haemoglobin
BP	Blood pressure
BMI	Body mass index
LDL	Low-density lipoprotein-cholesterol
HDL	High-density lipoprotein-cholesterol

## Appendix A. Search Strategies

PUBMED:

((“diabetes mellitus, type 2”[MeSH Terms] OR “type 2 diabetes mellitus”[tiab] OR “diabetes mellitus type 2”[tiab] OR “diabetes mellitus, type 2”[MeSH Terms] OR “type 2 diabetes mellitus”[tiab] OR (“non”[tiab] AND “insulin”[tiab] AND “dependent”[tiab] AND “diabetes”[tiab] AND “mellitus”[tiab]) OR “non insulin dependent diabetes mellitus”[tiab] OR “diabetes mellitus, type 2”[MeSH Terms] OR “type 2 diabetes mellitus”[tiab] OR (“diabetes”[tiab] AND “mellitus”[tiab] AND “type”[tiab] AND “ii”[tiab]) OR “diabetes mellitus type ii”[tiab] OR “niddm”[TIAB] OR “niddms”[TIAB] OR “hypoglycemic agents”[Pharmacological Action] OR “hypoglycemic agents”[MeSH Terms] OR (“hypoglycemic”[TIAB] AND “agents”[TIAB]) OR “hypoglycemic agents”[TIAB] OR (“hypoglycemic”[TIAB] AND “agent”[TIAB]) OR “hypoglycemic agent”[TIAB] OR “hypoglycaemics”[TIAB] OR “antihyperglycemic”[TIAB] OR “antihyperglycemics”[TIAB] OR (“hypoglycemic”[TIAB] AND “drug”[TIAB]) OR “hypoglycemic drug”[TIAB] OR (“antidiabetic”[TIAB] AND “drugs”[TIAB]) OR “antidiabetic drugs”[TIAB] OR (“antidiabetic”[TIAB] AND “agent”[TIAB]) OR “antidiabetic agent”[TIAB]) AND (“primary health care”[MeSH Terms] OR (“primary”[TIAB] AND “health”[TIAB] AND “care”[TIAB]) OR “primary health care”[TIAB] OR (“primary”[TIAB] AND “healthcare”[TIAB]) OR “primary healthcare”[TIAB] OR “ambulatory care”[MeSH Terms] OR (“ambulatory”[TIAB] AND “care”[TIAB]) OR “ambulatory care”[TIAB] OR “ambulatory care facilities”[MeSH Terms] OR (“ambulatory”[TIAB] AND “care”[TIAB] AND “facilities”[TIAB]) OR “ambulatory care facilities”[TIAB] OR (“ambulatory”[TIAB] AND “care”[TIAB] AND “facilities”[TIAB] AND “non”[TIAB] AND “hospital”[TIAB]) OR “ambulatory care facilities, non hospital”[TIAB] OR “primary nursing”[MeSH Terms] OR (“primary”[TIAB] AND “nursing”[TIAB]) OR “primary nursing”[TIAB] OR (“primary”[TIAB] AND “care”[TIAB] AND “nursing”[TIAB]) OR “primary care nursing”[TIAB] OR “primary care nursing”[MeSH Terms] OR (“primary”[TIAB] AND “care”[TIAB] AND “nursing”[TIAB]) OR (“nursing”[TIAB] AND “primary”[TIAB]) OR “nursing, primary”[TIAB] OR (“primary”[TIAB] AND “nursing”[TIAB] AND “care”[TIAB]) OR “primary nursing care”[TIAB] OR “physicians, primary care”[MeSH Terms] OR (“physicians”[TIAB] AND “primary”[TIAB] AND “care”[TIAB]) OR “primary care physicians”[TIAB] OR (“physicians”[TIAB] AND “primary”[TIAB] AND “care”[TIAB]) OR “physicians, primary care”[TIAB] OR (“physician”[TIAB] AND “primary”[TIAB] AND “care”[TIAB]) OR “physician, primary care”[TIAB])) AND ((COVID-19) OR “covid-19”[MeSH Terms] OR “covid-19”[TIAB] OR “covid19”[TIAB] OR “sars cov 2 infections”[TIAB] OR “sars-cov-2”[MeSH Terms] OR “sars-cov-2”[TIAB] OR “sars cov 2”[TIAB] OR “covid 19 pandemic”[TIAB]) → 192 results.

EMBASE

‘non insulin dependent diabetes mellitus’/exp OR ‘non insulin dependent diabetes mellitus’:ti,ab,kw OR ‘type 2 diabetes’:ti,ab,kw OR ‘diabetes type 2’:ti,ab,kw AND ‘primary care’:ti,ab,kw OR ‘primary medical care’/exp OR ‘primary medical care’:ti,ab,kw OR ‘primary health care’/exp OR ‘primary health care’:ti,ab,kw OR ‘general practitioner’/exp OR ‘general practitioner’:ti,ab,kw AND ‘coronavirus disease 2019’/exp OR ‘coronavirus disease 2019’:ti,ab,kw OR ‘covid 19’:ti,ab,kw → 342 results.

WEB OF SCIENCE

(“diabetes type 2” OR “type 2 diabetes” OR “non insulin dependent diabetes mellitus”) (Topic) and (“primary care” OR “primary medical care” OR “primary health care” OR “general practitioner”) (Topic) and (“covid-19” OR “coronavirus disease”) (Topic) → 140 results.
