# Supplementary material for: Exploring the Impact of the COVID-19 Pandemic on Patients with Type 2 Diabetes and Primary Care Utilisation: A Scoping Review
Source: Healthcare (Basel). 2026 Sep 1;14(17):2804. doi: 10.3390/healthcare14172804 (PMC13566578; doi:10.3390/healthcare14172804)
Supplement: Supplementary file 1 [file healthcare-14-02804-s001.zip › healthcare-4318002-supplementary.pdf]

Suplementary Materials—Detailed Summary of Included Studies

Table S1. Access to healthcare—direction of change

Author (Year)	Study population	Total PC visits	Face-to-face visits	Virtual consultation	Continuity of care	Emergency assistance	Specialist access	Care for complications	Referrals
**Coma et al. (2021) [12]**	n=>300,000	—	↓	—	↓	—	—	—	—
**Kowall et al. (2021) [13]**	n=32,399	—	—	—	—	—	—	—	—
**Carr et al. (2022) [14]**	n=618,161	↓	↓	↑	—	—	—	—	—
**Cuevas Fernández et al. (2022) [15]**	n=587	↓	↓	—	↓	—	—	—	—
**Di Gangi et al. (2022) [16]**	n=27,043	↓	—	—	↓	—	—	—	—
**Hooker et al. (2022)[17]**	n=42,942	↓	↓	↑	—	—	—	—	—
**Inglin et al. (2022) [18]**	n=11,458	↓	↓	↑	↔	↔	—	—	—
**Jug et al. (2022) [19]**	n=648	↓	↓	↑	↓	—	↑	—	↑

**Khmour et al. (2022) [20]**	n=17,413	↓	↓	↑	—	—	—	—	—
**Moin et al. (2022) [21]**	n=1,393,404	↓	↓	↑	↔	↓	↓	↓	—
**Strumann et al. (2022) [22]**	n=4,182	↓	—	—	↔	—	—	—	↔
**Cheng et al. (2023) [23]**	n=16,845	↓	↓	↑	↔	—	—	—	↓
**Koh et al. (2023) [24]**	n=644	—	↓	↑	↔	—	—	—	—
**Kowall et al. (2023) [25]**	n=21,747	↓	—	—	—	↓	↓	—	↓
**van den Berg et al. (2023) [26]**	n=182,048	↑	↑	↑	↔	—	—	—	—
**Cuevas Fernández et al. (2024) [27]**	n=3,543	↓	↓	↑	—	—	—	—	—
**Senthinathan et al. (2024) [28]**	n=39,401	↓	↓	↑	—	—	—	—	—
**van Grondelle et al. (2024) [29]**	n=607	↓	↔	↑	↔	—	—	—	—

**Freeman et al. (2025) [30]**	n=84,617	—	↓	↑	↓	—	↓	—	—
**Nielsen & Andersen (2025) [31]**	n=48,650	↑	↔	↑	↓	—	—	—	—
**Rijpkema et al. (2025) [32]**	n=15,247	↓	↓	—	↓	↑	↑	↑	—
**Weaver et al. (2025) [33]**	n=28,101	↑	↑	↑	—	↓	—	—	—
**Wong et al. (2025) [34]**	n=3,600	—	—	—	↔	—	—	—	—
**Yamamoto et al. (2025) [35]**	n=168,621	—	—	—	—	—	—	—	—
**Young et al. (2025) [36]**	n≈8,000	↔	↓	↑	↓	↔	↓	—	—
**Freeman et al. (2026) [37]**	n=159	—	↓	↑	↔	—	—	↔	—

↑ = Increased ↓ = Decreased ↔ = No significant change — = Not measured
Primary Care (PC)

Table S2. Disease management—direction of change

Author (Year)	Study population	T2D diagnosis	Follow-up	Requested controls	HbA1c request	HbA1c result	BP measurement	BP result	Weight/BMI measurement	Weight/BMI result	Laboratory determinations	Laboratory results	Eye examination	Foot examination	Controlled patients	Prescriptions
**Coma et al. (2021) [12]**	n=>300,000	↓	—	—	↓	↓	↓	↓	—	—	—	—	↓	↓	—	—
**Kowall et al. (2021) [13]**	n=32,399	—	—	—	—	↑	—	↑	—	↔	—	↑	—	—	↔	—
**Carr et al. (2022) [14]**	n=618,161	—	—	↓	↓	—	↓	—	↓	—	↓	—	↑↓	↑↓	—	↓
**Cuevas Fernández et al. (2022) [15]**	n=587	—	↓	↓	↓	↓	↓	↓	↓	↑	↓	↑	↓	—	↓	↔
**Di Gangi et al. (2022) [16]**	n=27,043	—	↓	↓	↓	↔	↓	↓	↓	↔	↓	↑	—	—	↓	↑
**Hooker et al. (2022)[17]**	n=42,942	—	↓	—	↓	↔	—	—	—	—	—	—	—	—	↓	—
**Inglin et al. (2022) [18]**	n=11,458	—	↓	—	—	—	—	—	—	—	↓	—	—	—	—	—
**Jug et al. (2022) [19]**	n=648	—	↓	—	↓	↔	↑	↔	↓	↔	↓	↓	↓	—	—	—
**Khmour et al. (2022) [20]**	n=17,413	—	—	—	—	↔	—	—	—	↔	—	—	—	—	↔	—

**Moin et al. (2022) [21]**	n=1,393,404	–	–	↓	↓	–	–	–	–	–	↓	–	↓	↑↓	–	↔
**Strumann et al. (2022) [22]**	n=4,182	–	–	–	–	↔	–	↑	–	↔	–	–	–	–	↔	–
**Cheng et al. (2023) [23]**	n=16,845	–	–	↓	↓	↔	↓	↔	↓	↔	↓	↔	–	–	↔	–
**Koh et al. (2023) [24]**	n=644	–	–	–	–	↔	–	–	–	–	–	–	–	–	–	–
**Kowall et al. (2023) [25]**	n=21,747	↓	↓	↓	↓	↑	↔	↔	–	↔	–	↑	–	–	↓	↑
**van den Berg et al. (2023) [26]**	n=182,048	–	↓	↓	↓	↑	↓	↑	↓	↓	↓	↓	–	–	↓	–
**Cuevas Fernández et al. (2024) [27]**	n=3,543	–	–	–	↓	↓	–	–	–	–	–	–	–	–	↓	–
**Senthinathan et al. (2024) [28]**	n=39,401	–	–	–	↓	↔	↓	↔	–	–	–	–	–	–	–	–
**van Grondelle et al. (2024) [29]**	n=607	–	↓	↓	↓	↑	↓	↑	↓	↔	↓	↔	–	–	↔	–
**Freeman et al. (2025) [30]**	n=84,617	–	↓	–	↓	↔	↓	↔	–	–	↓	–	–	–	↔	↔

**Nielsen & Andersen (2025) [31]**	n=48,650	↔	—	—	—	—	—	—	—	—	—	—	—	—	—	—
**Rijpkema et al. (2025) [32]**	n=15,247	—	↓	—	—	—	—	—	—	—	—	—	—	—	—	—
**Weaver et al. (2025) [33]**	n=28,101	—	—	—	—	—	—	—	—	—	—	—	—	—	—	↑
**Wong et al. (2025) [34]**	n=3,600	—	—	—	—	↔	—	↔	↔	↔	—	—	—	—	↔	—
**Yamamoto et al. (2025) [35]**	n=168,621	—	—	—	—	↑	—	↑	—	↓	—	—	—	—	↓	↑
**Young et al. (2025) [36]**	n≈8,000	—	—	—	↔	—	—	—	—	—	—	—	—	—	—	↔
**Freeman et al. (2026) [37]**	n=159	—	—	—	↔	↔	↓	↔	↓	↑↓	↔	↔	↔	↔	—	—

↑ = Increased ↓ = Decreased ↔ = No significant change — = Not measured

Type 2 diabetes (T2D); glycated haemoglobin (HbA1c); blood pressure (BP); body mass index (BMI)
